# Inebriety and the Use of Opium

**Published:** 1882-11

**Authors:** 


### Inebriety and the Use of Opium.

In a review of thirty-five cases of *inebriety* and the use of opium, admitted to Walnut Lodge, Hartford, Conn., during the year 1878, discharged within the year, and followed up for four and a half years since, Dr. Crothers specially notes the following facts: Heredity was a prominent factor in causation, traceable in nearly all the cases. In many there was also a special exciting cause in the shape of mental or bodily strain. In a large proportion of cases, when inebriety begins it follows a more or less regular order or course, which can be traced and anticipated in study and treatment. The essentials of treatment are, briefly, the removal of all exciting causes; building up the general strength and vigor of the organism, physically and morally; the withholding of alcohol as absolutely as possible, all restraint being adapted to the special needs of each case; these means being used for periods of not less than from one to three years. The results, after treatment in the asylum for from thirty-four days to six months, averaging four months to each patient, have been as follows: *Nine*, including two who have died, remained temperate. *Five* relapsed once, but are now temperate. *Five* relapsed twice or more, at long intervals, and are now well. *Seven* relapsed, and are still drinking. *Four* relapsed, and died or became insane. *One* died under treatment. In the remaining *four* of the thirty-five cases no history has been obtained since they were discharged "greatly benefited." Dr. Crothers states that all these patients had developed a low grade of chronicity, and had exhausted every means of treatment before coming to the asylum as a last resort. He thinks that the results of treatment to-day, with the worst cases and the crudest means and methods of restoration, only faintly indicate the possibility of cure in the future. The restoration of seven in thirty-one cases, after a period of four years and more, is an unmistakable sign of the eminent curability of inebriety, with better means and larger knowledge.—*N. Y. Med. Jour. and Obst. Review.*

